# Reframing NCDs? An analysis of current debates

**DOI:** 10.1080/16549716.2019.1641043

**Published:** 2019-07-31

**Authors:** Kafui Adjaye-Gbewonyo, Megan Vaughan

**Affiliations:** Institute of Advanced Studies, University College London, London, UK

**Keywords:** Non-communicable diseases, social determinants of health, epidemiology, health transitions

## Abstract

There have been many debates in recent years as to whether the communicable disease versus non-communicable disease (NCD) division is a meaningful one in disease classification. Several critiques have been raised about the framing of NCDs, regarding not only the prominent role that infections play in the aetiology of NCDs, but also the communicability of many social determinants of NCDs and the individualistic, ‘lifestyle’ framing of NCDs that tends to focus on health behaviours to the neglect of socio-political, environmental, and structural determinants of health. In this paper, we give a historical overview of the usage of the NCD terminology and analyse some of the recent debates regarding the naming and framing of NCDs. We argue that a lack of reflection on the assumptions underlying the naming and framing of NCDs may lead to the collection of insufficient epidemiological data, the development of inappropriate interventions and the provision of inadequate care. Work in social epidemiology, health promotion, medical anthropology, demography, and other fields may provide insights into the ways in which efforts targeting NCDs may be reframed to improve impact and efficacy. In addition, concepts such as multimorbidity and syndemics, frameworks such as ecosocial theory and approaches based in the social sciences may provide a way forward in the conceptualization of disease.

## Background

‘Non-communicable diseases (NCDs)’ seems to have become a catchphrase in global health. While its definition is not universally agreed upon, according the Medical Subject Headings (MeSH) definition for NCDs, which was just introduced in 2018, NCDs are ‘Diseases of long duration and generally slow progression’. The MeSH definition goes on to state that, ‘The four main types of noncommunicable diseases are cardiovascular diseases (e.g. heart attacks and stroke), cancer, chronic respiratory diseases (e.g. chronic obstructive pulmonary disease and asthma) and diabetes mellitus’ [1]. This paper seeks to question and problematize the concept of NCDs by reflecting on the assumptions behind this category and how they might affect research and practice. The goal of this paper is not necessarily to argue for abandoning the phrase ‘NCDs’ or to suggest an alternative, but rather to analyse some of the recent debates around the framing of NCDs and the underlying issues these debates point to. Our main argument is that the public health community may need to be more reflexive and critical of the sometimes inadequate and artificial divisions used in classifying diseases and how these divisions may limit the ways in which we research and intervene in health. Concepts such as multimorbidity and syndemics, frameworks such as ecosocial theory and social-science-based approaches may provide a more useful way to understand the epidemiologic challenges of our time. But first, it may be useful to look briefly at the history of the term’s usage in the scientific literature.

### NCDs: usage over time

The concept of what we call NCDs largely developed from the notion of ‘chronic disease’, and the two phrases often continue to be used interchangeably. The phrase ‘chronic disease’ has a longer history of common use, with Weisz noting that it dates back to the Greco Roman period [2]. Such diseases, whether infectious or not, were historically viewed as incurable. However, it is in the twentieth century that the concepts of chronic disease and chronic illness began to acquire the meanings and significance to which we assign them today, often connoting non-infectious conditions that typically require chronic care and are frequently, though not exclusively, associated with aging. In the 1920s, chronic diseases became a regular category in *Index Medicus*, with ‘chronic illness’ being added in 1947 [2,3]. In 1935, the U.S. undertook its first National Health Survey of Chronic Illness and Disability, which later became the National Health Interview Survey. Weisz notes that the concept of chronic disease was initially largely an American concern until the 1960s when it began to be adopted by other countries [2]. In the post-World-War-II era, British epidemiologists such as Bradford Hill, Doll and McKeown also advanced the study of chronic disease with their research on smoking, cardiovascular disease and cancer [4]. The study and control of chronic disease in the last century greatly influenced the fields of epidemiology and public health, simultaneously advancing the more individualistic biomedical and lifestyle models of disease [2,5–7]. For example, it was from the Framingham Heart Study, that the phrase ‘risk factor’ was coined in 1961 [6].

Thus, a distinction was made in terms of the duration of disease where many infectious conditions were viewed as being acute, resulting in either death or recovery, while many non-infectious diseases were seen as incurable and chronic [2]. However, this distinction has not always been so clear cut as many have noted [2,8], and it can become problematic in its focus on biomedical constructs without recognition of social factors that may impact the curability and chronicity of disease, such as access to care [9].

In recent years, the phrase ‘NCDs’ has often been applied, with a more global outlook. In the 1970s, NCDs became part of the global health agenda with efforts of the World Health Organisation (WHO), such as through their Noncommunicable Disease Division, and in the 1980s this focus expanded to low- and middle-income countries (LMICs) with the WHO’s INTERHEALTH programme [2,5]. In addition, the landmark Global Burden of Disease (GBD) Studies, initially published for the World Bank in 1993 and subsequently conducted with the participation of the WHO, have helped to familiarise stakeholders with the term ‘NCDs’ and with the significance of their disease burden. The GBD studies provide global estimates of the burden of various diseases and risk factors and classify diseases and conditions into three broad categories at Level 1. These are: (1) Communicable, maternal, perinatal/neonatal and nutritional conditions; (2) non-communicable diseases; and (3) injuries. These broad categories are further broken down at Levels 2 and 3 following the International Classification of Diseases [10–12]. The significance of these studies to global health and policy has arguably helped to solidify a standard classification of diseases. Moreover, recognition of the fact that many infectious diseases, like tuberculosis, are also chronic, and that with improved treatment, diseases such as AIDS have also become chronic, has spurred wider use of the term ‘NCDs’ to further distinguish these ‘non-infectious’ conditions from other diseases that are also chronic in nature but viewed as infectious.

To illustrate how the use of the phrase ‘NCDs’ has evolved over time, we conducted a search for the phrase ‘NCDs’ in PubMed using the search terms in Box 1 below. The search produced over 8000 results, the earliest of which is a Portuguese-language paper from Brazil published in 1961 by D. Wilson and entitled, ‘The considerable increase of non-communicable diseases as causes of death in the city of Sao Paulo from 1900 to 1960’ [13].

**Box 1.** Pubmed search terms for NCDs.

“noncommunicable diseases“[MeSH Terms] OR (“noncommunicable“[All Fields] AND “diseases”[All Fields]) OR ”noncommunicable diseases”[All Fields] OR ”non communicable diseases”[All Fields] (Search conducted 12 November 2018)

This was followed by several papers in Russian from the late 1960s on animal non-communicable diseases [14–17]. The next references relating to human disease were a 1972 article in German on ‘Activities of the World Health Organization in the field of non-communicable diseases’ [18], followed by a 1977 Spanish-language paper from Mexico, ‘Control of noncommunicable diseases in the primary level of health care’ [19].

**Figure 1.**
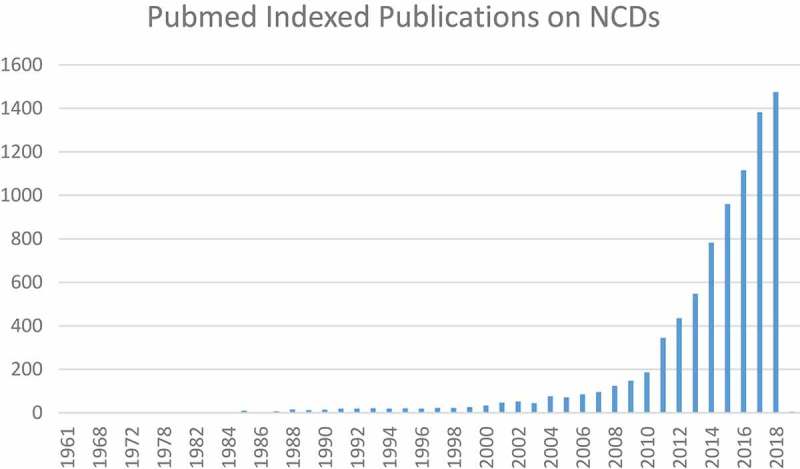
Pubmed indexed publications on NCDs by year.

Thus, many of the early references to NCDs that are indexed in PubMed come from non-English-language uses of the terminology, and it is not until mid-1977 that an English-language paper from the *Canadian Journal of Public Health* is documented in PubMed using the phrase in its title [20]. Publications documented in PubMed using the phrase NCDs have steadily increased over time, taking off particularly in the second decade of the 2000s (Figure 1).

Given that PubMed may not contain a complete listing of scholarly publications on NCDs, we conducted similar searches in Google Scholar and Web of Science for comparison. In Google Scholar, a search of the terms ‘non-communicable disease(s)’ or ‘noncommunicable disease(s)’ retrieves an earlier publication in the *Journal of Chronic Diseases* in its initial year of publication in 1955 [2,21]. This paper additionally cites the 1954 article, ‘Epidemiology in Noncommunicable Disease,’ by Gilliam [22]. Web of Science searches produce largely similar results beginning with the 1954 paper by Gilliam followed by citations from the early 1970s, which include reviews for the books *Communicable and Noncommunicable Diseases* by Jones, Shainberg and Byer and *Epidemiology of Non-Communicable Disease* by Acheson. Thus, it is evident from these three searches that certainly by the mid-twentieth century, the phrase ‘NCDs’ had begun to be used in academic circles and that its usage has grown in recent years.

The dramatic increase in publications on NCDs illustrated in Figure 1 largely mirrors events occurring in the global health arena over the past decade. In 2008, the WHO’s annual *World Health Statistics* report highlighted the global shift from infectious to non-communicable diseases [23], and the organisation released its *2008–2013 Action Plan for the Global Strategy for the Prevention and Control of Noncommunicable Diseases* [24]. In 2011, the United Nations (UN) held its first High-Level Meeting on NCDs with subsequent meetings in July 2014 and most recently in September 2018.

### Framing NCDs

The 2008 WHO report, the UN High-Level Meetings on NCDs and the activism around these events from organizations such as the NCD Alliance [25], helped to put NCDs on the global health map, increasing recognition of the term and the problem. The drive to increase global action on NCDs can in some ways be seen as a reaction to the intense focus on the communicable, maternal, neonatal and nutritional group of diseases in the Millennium Development Goals (MDGs) which helped to shape the global health agenda in the early 2000s [26]. For example, some of the major global health funders of the early twenty-first century – the Global Fund to Fight AIDS, Tuberculosis & Malaria; the U.S. President’s Emergency Plan for AIDS Relief; the Bill & Melinda Gates Foundation; UNAIDS; Clinton Health Access Initiative, the Global Alliance for Vaccines and Immunization – were largely motivated in response to the health-related MDGs and other global commitments which focused on undernutrition, maternal and child health, malaria and AIDS [27,28]. For those working in NCD areas, this focus was seen to neglect NCDs, which were known to be major global killers and also on the rise. Thus, the advocacy work and research to recognise the NCD ‘epidemic’ helped to ensure that NCDs were included in the Sustainable Development Goals (SDGs) with SDG 3, ‘Ensure healthy lives and promote well-being for all at all ages,’ encompassing not just nutritional, maternal, neonatal and infectious diseases but also NCDs, injuries and neglected tropical diseases [26]. Nevertheless, the very act of emphasizing the distinction between NCDs and infectious and other diseases in order to raise awareness, may also have contributed to some of the controversies and complications that we will discuss below.

The framing of NCDs has historically been dominated by several related frameworks. These are the biomedical model and lifestyle frameworks and the theory of epidemiological transition. The biomedical model, which characterized epidemiology in the twentieth century, centres on biological and physical causes and mechanisms of disease. It focuses on individuals and is reductionist in nature, viewing the whole as merely the sum of its parts [6]. In the context of NCDs, this framing has put an emphasis on risk factors, including genetic and biological risk factors such as age, race/ethnicity, sex, family history, et cetera. The ‘lifestyle’ framing similarly puts emphasis on individuals, with a focus on health behaviours which are often implicitly assumed to be lifestyle ‘choices’ [6]. When applied to NCDs, the ‘lifestyle’ framing has tended to focus on the ‘big four’ conditions described in the MeSH definition of NCDs (cancer, cardiovascular diseases, diabetes and chronic respiratory diseases) and four major modifiable risk factors of tobacco, alcohol, physical inactivity and unhealthy diets.

The theory of epidemiological transition has also played a role in the framing of NCDs. First described by Abdul Omran in 1971, the theory put forward a model in which societies transition from having an epidemiologic and mortality profile marked by undernutrition and infectious disease (age of pestilence and famine), to a period in which such conditions decline, and finally toward a stage dominated by chronic and non-communicable diseases as life expectancies, economic prosperity and industrialisation increase (age of degenerative and man-made diseases) [29–31]. This has been used to explain and predict trends of increasing rates of NCDs and declines in infectious disease globally, particularly in LMICs. It has often also been interpreted to suggest that NCDs are associated with affluence and economic well-being because they are expected to become more prevalent as nations industrialise and develop economically. Through all this, a distinction has been made between infectious diseases and NCDs as being separate across time and space.

### Reframing NCDs

The framing of NCDs and what is being overlooked have been growing topics of concern, however. These concerns have largely been motivated by desires to increase attention to and resources for certain types of NCDs or NCDs generally. Building on issues raised in a 2011 conference on the endemic NCDs of the poor [32], in 2015 physicians Gene Bukhman and Ana Mocumbi launched a Lancet Commission on Reframing NCDs and Injuries for the Poorest Billion (Lancet NCDI Poverty Commission), whose goal was to bring an equity lens to the NCD agenda by highlighting the types of NCDs linked to poverty–conditions that are often neglected by the framing of NCDs as ‘lifestyle’ or behavioural diseases [33]. Bukhman and colleagues argue that the NCDs that have been prevalent in some of the poorest communities worldwide instead include conditions such as rheumatic heart disease and Burkitt’s Lymphoma – which have infectious and environmental causes rather than behavioural causes – as well as congenital and genetic disorders and mental illness and injuries. Indeed, the biomedical and lifestyle/behaviour framing of NCDs has often pushed environmental and structural determinants of health to the background [33–35]. A recent article about global environmental change and NCDs noted that not only have environmental determinants of NCDs been overlooked in the past by the WHO and others, but also that even the recent debates on the framing of NCDs have continued to ignore global environmental change [35]. It was only last year that air pollution, for example, was added as a risk factor to the WHO framework on NCDs [36].

Complementary efforts to re-examine notions of epidemiological transition and NCDs from a historical perspective have been underway with the ‘Chronic Disease in Sub-Saharan Africa: A Critical History of an ‘Epidemiological Transition’’ project led by historian Megan Vaughan at University College London [37]. This project seeks to challenge the idea of transition by examining local histories and conditions; it also seeks to highlight the important interactions between infectious diseases and NCDs, interactions that are often overlooked when dividing these conditions into distinct categories or assigning them to different points in time.

Public health specialists Abel and McQueen have also critiqued the theoretical framing of NCDs [38]. They argue that while theories of NCDs are often not made explicit, the epidemiological basis used to study NCDs has implicitly led to a focus on individuals to the exclusion of contextual factors; the social is essentially only studied to the extent that it can be causally linked to biological disease. The authors argue that this framing has also led to a focus on risk, risk factors and disease rather than on health and health resources, making individuals and populations passive recipients of health-damaging exposures rather than co-producers of health. In addition, they argue that the framing of NCDs typically relies on reductionist one-way, linear causal thinking, often ignoring complexity. The authors advocate instead for a social-science-based and health-promotion-focused framework for NCDs, a point that will be returned to later in this article.

### Recent debates on the NCD moniker

Along similar lines, there have also been a number of recent debates as to whether the name ‘NCD’ itself is even an effective or useful one. In the early- to mid- 2000s, one such debate appeared in the *Journal of Epidemiology and Community Health*. Ackland and colleagues called for returning to the phrase ‘chronic disease’ in place of ‘NCD’, arguing that the societal determinants of health behaviours are communicable – they are marketed, communicated and passed down through families and communities along social gradients – and thus should be treated as social ‘vectors’ and as the focus of interventions. The authors hoped to place a sense of urgency and motivate ‘upstream’ thinking on chronic disease and added that the phrase ‘transmissible chronic disease’ could be used to emphasize these social determinants [39,40]. Unwin and colleagues disagreed with their proposal, however, noting that it confused a classification based on causes (communicable and non-communicable) with one based on effects (acute versus chronic), overlooking the fact that many chronic conditions were communicable and many non-communicable conditions were acute [40].

This debate highlighted several themes that would be returned to nearly a decade later in the 2010s. One work that revisited such themes was the *Global Handbook on Noncommunicable Diseases and Health Promotion* [7]. In the introduction to the volume, editor David McQueen lamented the ‘procrustean’ nature of the NCD terminology which has been a subject of debate in public health. The shortcomings of this terminology included the implied non-infectious aetiology of NCDs, despite the fact than many NCDs have infection-related aetiologies. While most public health researchers and practitioners do recognise the limitations of the NCD terminology, he noted that it has become a globally accepted term, in part because of its use by the WHO [41].

In his 2014 book, *Infections, Chronic Disease and the Epidemiological Transition*, demographer Alexander Mercer proposed that when classifying diseases, a combination of three dimensions be considered, while simultaneously allowing for potentially unknown factors in these dimensions [8]. One dimension is the cause of the disease (infectious agent – known, possible or none); the second is the effect of the condition (acute or chronic); and the third is communicability. Thus, diseases could be viewed as acute and communicable with known infectious causes (e.g. measles), chronic and communicable with known infectious causes (e.g. tuberculosis), chronic with non-infectious aetiology and not communicable (e.g. genetic disorders, though one could in theory argue that genetic transmission is a form of communicability), et cetera. This model for classifying disease is perhaps more precise than our current classification system though a bit more cumbersome as well.

In late 2016, a discussion was started by Justin Zaman in the ‘Non-Communicable Diseases’ online community at GHDonline, a platform of medical and health professionals that forms part of the Global Health Delivery project [42]. He argued that far from being ‘non-communicable’, NCDs were in fact ‘spreading’ from urban to rural areas with globalization and ‘development’. In the responses that followed, some agreed with Zaman, while others commented that conditions such as hypertension are also high in communities that have not yet adopted dietary and lifestyle changes characteristic of ‘economic development’. Yet other responses contended that the NCD versus infectious disease distinction is relevant and is borne out by the fact that one says that a person is ‘affected’ by an NCD and not ‘infected’ by it, or that while infections such as HPV can cause NCDs, the cancers themselves are not infectious diseases.

In 2017, this debate was taken to the *Lancet Global Health* by Luke Allen and Andrea Feigl after a commentary by *Lancet* editor Richard Horton lamented why the global health community has not effectively responded to NCDs and suggested that framing NCDs in terms of fear and security risks, in the way that infectious diseases have been framed, may help [43,44]. This lack of charisma for NCDs compared to infectious diseases has also been highlighted by geographer Clare Herrick [45]. Allen & Feigl responded to Horton’s commentary by arguing that classifying NCDs in terms of what they are not deprives them of the urgency needed to tackle the epidemic and focuses on the individual-level rather than societal-level drivers of these conditions. They solicited suggestions from the *Lancet* readership as well as the GHDonline community to re-name NCDs. Several authors responded to their call with commentaries in *Lancet Global Health*. Some of the names that were put forward included, ‘lifelong diseases’ and ‘biosocial and development diseases’, among others [35].

Synthesizing the feedback they received, Allen and Feigl eventually proposed the phrase ‘socially-transmitted conditions (STCs)’ as an alternative to ‘NCDs’ in order to draw attention to the shared commercial and social determinants of these conditions [46]. However, in follow-up discussions, Cavalin and Lescoat argued that this phrase may inadvertently lead to the suggestion that infectious diseases do not share some of these social determinants [47]. Nevertheless, on the GHDonline community, some feedback on this debate suggested that worrying about the NCD name may be of little relevance to communities and that energies should instead be devoted to taking action to prevent them [48].

However, the discussion about the framing and naming of NCDs has not gone away. In a recent article, Blundell and Hine argued for abandoning the NCD label for many of the reasons noted before, including that it has become a catch-all and consequently meaningless label for a wide variety of conditions, that there is a link between infections and many NCDs, and that many of the social risk factors for NCDs are also ‘communicated’ [49]. They argued instead for a focus on the ‘human-made’ nature of NCDs and attention to the social determinants of health. Similar arguments about the communicability of NCDs and their determinants through biosocial contagion; the conflation of ‘infectiousness’ and ‘communicability’; the need to target the macro-level ‘vectors of transmission’ of NCDs; and the ways in which the framing of NCDs as ‘lifestyle’ diseases has driven epidemiologists to neglect other research questions, have also been made by medical anthropologists Seeberg and Meinert [34].

The NCD terminology was also a topic of discussion during a recent workshop entitled, ‘Africa and the Epidemiological Imagination.’ Issues such as comorbidity and multimorbidity, interactions between infectious and ‘non-communicable’ diseases, commercial determinants of health, et cetera were debated. Amy Moran-Thomas discussed her alternative notion of ‘paracommunicability’, and deliberations generally led to the conclusion that ‘the frame of ‘noncommunicable’ obscured more than it illuminated,’ as summarized by Mika and Vaughan [50,51]. Moreover, it was noted that, more than just being an issue of semantics, the framing and redefining of NCDs in terms of so-called ‘modifiable’ behavioural risk factors has shaped what and how epidemiological data on these conditions are collected and consequently how interventions to prevent them are approached [51].

In the case of Malawi, for example, Vaughan describes how the framing of NCDs there became driven by a reductive, risk factor approach that focused on ‘metabolic syndrome’ or ‘metabolic disorder’ [51]. This emphasized individual behaviour and ‘lifestyle’ and overlooked other complexities of metabolism including historical processes, life-course effects of early-life undernutrition and poverty, and the role of infection. Rather, Vaughan argues that the WHO framing of NCDs as inherently ‘non-communicable’ and driven by four modifiable risk factors led to the collection of epidemiological data on NCDs in Malawi based on the STEP-Wise Approach to Disease Risk Surveillance (STEPS) survey, a standardised WHO survey that has been implemented in many countries. The STEPS survey consists of a questionnaire collecting socio-demographic information as well as core data on tobacco and alcohol use, fruit and vegetable consumption and physical activity. In addition, blood pressure and anthropometric measurements and biochemical measurements of blood glucose and cholesterol are collected. The STEPS was conducted in Malawi in 2009. Vaughan contends that this framing of NCDs may have led to a missed opportunity to gather data on other types of NCDs (apart from the behavioural and metabolic conditions); on the role of infection in NCDs, including the HIV/AIDS epidemic which affected Malawi; on environmental exposures; as well as on the ongoing problem of undernutrition which has also been linked to NCDs [51].

Similarly, work by Kwan and colleagues has shown that the cardiovascular diseases among the world’s poor are often not due to ischemia from ‘lifestyle’ causes, but are much more diverse in nature, including a significant proportion due to infection, as well as congenital conditions and other factors related to poverty and environment [52]. Heart failure registries from Haiti and several African countries bear this out [52]. Thus, the danger in the restrictive framing and classification of NCDs in a way which downplays the links to infectious, environmental, social and societal causes, has the potential to contribute to not only incomplete data collection but also to restrictive healthcare interventions, further entrenching vertical programming in health systems, for example.

## Themes: complex aetiologies and multimorbidities

One theme that seems to emerge from the debates is that of aetiology. While terms such as ‘infectious diseases’ point directly toward a step in the aetiological pathway of these conditions (namely infections), NCDs are defined in terms of what they are not, as highlighted by Allen and Feigl [44]. Part of the complication is that they are a diverse group encompassing not only the ‘big four’ conditions, but also mental illnesses (which were recently added to the WHO NCD framework [36]), genetic and congenital disorders, autoimmune disorders and musculoskeletal conditions, among others [46,53]. Moreover, many NCDs have very complex aetiologies [4], including gene-environment interactions and epigenetic changes. Most can be linked to exposure to either infectious agents (human papillomavirus, strep, hepatitis C), environmental toxins and chemicals (air pollution, tobacco smoke, asbestos, alcohol), injury (physical injury, radiation) or psychosocial exposures (stress), all of which can lead to chronic inflammatory processes, thereby causing disease [54]. The aetiological periods are often lengthy, with many NCDs developing over the life-course or having intergenerational influences, as work on the fetal and developmental origins of disease has shown [55]. Even what are considered the modifiable metabolic risk factors can be precipitated by various factors including infections and undernutrition.

In addition, the traditional NCD distinction and epidemiological transition narrative separating NCDs from infectious diseases across time and place seem to gloss over multimorbidities on both an individual and a societal level. Far from belonging to separate eras, communicable, maternal, neonatal and nutritional diseases often co-occur with NCDs, particularly in LMICs, thereby violating the assumptions of a separate ‘age of pestilence and famine’ and ‘age of degenerative and man-made disease,’ as described by the theory of epidemiologic transition [4,30,31]. This co-occurrence is commonly referred to as the ‘double burden of disease’ [29]. Some have even referred to a ‘triple burden of disease’, adding the ailments of globalization, such as pandemics and climate change, into the mix [56]. Further, not only do NCDs and communicable, maternal, neonatal and nutritional diseases often present and co-exist within the same society, but they often co-exist within the same individual. Interest in this area has rightfully been growing, and the Global Alliance for Chronic Disease recently put out a research statement highlighting the need to consider multimorbidity in research and healthcare [57].

## Themes: socio-political determinants of health in historical perspective

One of the other main issues that the debates around the framing and naming of NCDs have highlighted is a dissatisfaction with the individualistic biomedical model of disease to the neglect of social, behavioural, political and environmental factors [47,49,58]. These factors include socioeconomic conditions, inequality, food systems and industries, policies and governance, rights, living and working conditions, et cetera [6]. Allen and Feigl argued that addressing these social drivers when it comes to NCDs is a good starting point that could lead to upstream thinking in other areas [58]. Nevertheless, upstream thinking in epidemiology and health is not necessarily novel. Since the mid- to late-1900s, the field of social epidemiology has tried to draw attention to these upstream social and societal (including political and commercial) drivers of health – the ‘causes of the causes’ [6]. Often drawing on social-ecological models, social epidemiology attempts to examine the multiple levels at which health is shaped, from the individual to the macro-level. From this perspective, all disease has social causes or implications. However, even before the birth of this field of public health, the history of epidemiological inquiry had an upstream focus. Louis-Rene Villermé, Rudolf Virchow, Friedrich Engels and others examined morbidity and mortality among the urban and industrial poor in Europe in the 1800s [4,6]. They related ill health to issues of class, political economy, industrialisation, and the social and environmental conditions in which many people were forced to live and work. Disease was therefore societally-produced and the work of these scholars was the foundation of and precursor to what Krieger considers the socio-political theories of disease distribution [6].

Similarly, Richard Horton in a recent commentary reminded the global health community of its socio-political origins [59]. He argued that global health is concerned with the national and transnational forces – including poverty, exploitation, violence, power, and oppression – that harm health, and that it was decolonization that birthed the global health movement. In particular, Horton credits post-colonial writer-clinician, Frantz Fanon with writing the first works of the global health movement linking colonization and medicine.

Thus, while public health may have been seen as more of a political science in the 19^th^ century, global health and epidemiology seem to have lost track of their roots in socio-political determinants of health with the advent of biomedical and lifestyle models of health in the 20^th^ century [6,7]. This has led to the individualistic and disease-based ‘black box’ epidemiological approaches that have dominated in past century with regard to NCDs and other conditions [4]. Despite a resurgence in attention to social determinants of health in recent decades, as exemplified by the work of Michael Marmot and others, many would agree that additional work is needed to expand non-biomedical perspectives in the NCD narrative.

## Where to go from here …

### Naming

We acknowledge that given how embedded the NCD terminology has become in global health and that the public has also begun to familiarise itself with this term, it may be unproductive at this point to argue for a new name. Although we applaud some of the previous efforts, it seems a strong alternative has not yet emerged. However, the framing and frameworks used to address NCDs need reconfiguration. Already, as we have shown above, there are numerous attempts to reframe the NCD narrative, and we feel that additional work in this area would be beneficial to disease prevention and control. Moreover, without first addressing our underlying frameworks for NCDs, any new names adopted may also run the risk of assuming the same meanings and uses that the phrase ‘NCDs’ currently has. Thus, we agree with Herrick’s assertion in her recently published think piece that the failure of NCDs to galvanise action is not just due to the name but also to deeper issues [60].

### Framing and frameworks

**Figure 2.**
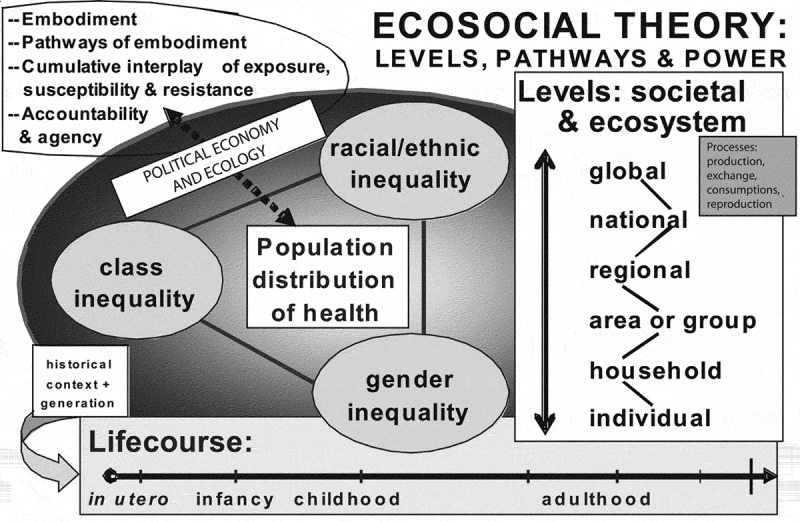
A heuristic diagram for guiding ecosocial analyses of disease distribution, population health, and health inequities, reproduced from Krieger [1^61^, p. 224]  with permission from the American Public Health Association.

We believe that there are a number of frameworks that may help to better address and frame NCDs. One is Krieger’s ecosocial theory. Ecosocial theory incorporates political economy, life-course and historical factors, accountability for social disparities in health and for research on these inequalities, and social and biological processes at different levels and spatiotemporal scales. It uses the concept of ‘embodiment’ to explain how we biologically incorporate the material and social worlds we inhabit [6] (see Figure 2).

The concept of multimorbidity introduced earlier may also help to strengthen our response to disease by allowing us to consider the co-occurrence of and interactions between multiple conditions within individuals, including interactions between diseases normally placed in different categories. On a population level, a related concept that has been gaining increasing attention is that of ‘syndemics’. Syndemic theory posits that multiple epidemics, conditions and social problems can arise in populations due to social conditions and can interact synergistically within these populations, creating an excess burden of disease that is greater than the sum of the conditions individually. These conditions are socially-patterned, tending to cluster in populations that are impoverished or marginalized. Rather than viewing diseases or epidemics as discrete conditions, syndemic theory views them in relation to and mutually reinforcing social and environmental conditions [34,62,63]. For example, a report was recently published in *The Lancet* on the global syndemic of obesity, undernutrition and climate change [64]. The commissioners put forward recommendations to address the epidemics of obesity, undernutrition and climate change, which they argued share common societal causes and co-occur and interact in place and time, affecting people worldwide in a global syndemic.

We believe that these concepts of multimorbidity and syndemics and the use of an ecosocial framework may be particularly useful starting points for the health professions to start reframing the ways in which we think about and act on disease prevention and control. Ideally, data collection and interventions for NCDs would attempt to follow an ecosocial framework and consider multimorbidity and syndemics by including not only chronic conditions, health behaviours, and individual and household socioeconomic status, but also infectious illnesses and other comorbidities, undernutrition, genetic and biological factors, psychosocial factors, and neighbourhood and environmental characteristics. In addition, these data and interventions would incorporate life-course perspectives from childhood to adulthood, as well as intergenerationally. Geographical information at multiple scales would be considered, allowing one to link contextual factors to individuals by time and place. This also means that resources should be devoted to measuring and monitoring various structural and contextual factors such as polices that may impact well-being. However, although this type of data collection and intervention design may be ideal, it often turns out to be less straightforward in practice. Limitations arise, including optimizing survey/interview length to collect rich data without increasing non-response; research fatigue in communities that are often studied or under surveillance; respondent privacy in ever more detailed data; and limited financial resources for extensive and large-scale data collection and interventions over the long-term. How to tackle these challenges in innovative ways that will allow us to effectively study epidemiology and respond accordingly should be a priority for global health.

One final recommendation to consider comes from Abel and McQueen who argue for a social-science-based theory of NCD causation that is driven from the social rather than from the biomedical [38]. Going beyond social epidemiological methods that nonetheless start from and centre on disease outcomes, they contend that social context should be the primary concern and target of interventions. In other words, they argue that even social epidemiology starts by identifying diseases and conditions (cancer, violence, etc.) and then attempts to understand what social factors determine those conditions and which can be changed. Abel and McQueen recommend starting first from the social context (perhaps neighbourhoods, policy environments, etc.) and making those, rather than the disease outcomes, the primary factors of interest and targets of action. The public health field has begun to move in those directions (again), with programmes, courses and centres focused on issues such as neighbourhoods and health, urban health, the built environment, health and happiness, social policy or health and society emerging at various institutions. Nevertheless, additional movement in this direction may be a welcome improvement to the study of health and illness.

McQueen and Abel further contend that, rather than being data-driven, the approach to NCDs should be social science based, incorporating notions of complexity and dynamic associations, while integrating epidemiological knowledge [38]. McQueen also argues that the subfield of health promotion within public health is one that could help to improve work in NCDs by addressing the broad issues of social context that anthropologists and political and social scientists often focus on [41]. Health promotion, he argues, emphasizes social action, equity, social justice, and health and human rights in order to promote health and not merely to prevent disease. It should be noted that health promotion as a field is not without criticism [65,66], and as McQueen acknowledges, also needs more theoretical exposition [41]. Nevertheless, there may be lessons to be learned from health promotion in the battle against NCDs.

## Conclusions

When it comes to naming and classifying NCDs, the optimal solution may still be up for debate. However, it does seem clear that the current framing is inadequate. Moreover, the debates discussed in this paper highlight themes that are not unique to NCDs but touch on how we think about disease and illness more generally. The debates on NCDs reveal a desire for solutions that give renewed attention to the socio-structural, political and environmental determinants of health and for the incorporation of interdisciplinary perspectives, including those emanating from the social sciences. In summary, we believe that it is critical to question the ways in which we frame and name NCDs and be critically aware of the assumptions that underlie our typological systems. Doing so may improve the epidemiological data we collect, the methods we use, the resources we summon, and the interventions we propose to combat this health issue. The concepts of multimorbidity and syndemics, frameworks such as ecosocial theory, and social-science-based approaches may provide promising avenues for reframing NCDs.

## Paper context

Health professionals and scholars from various fields have expressed dissatisfaction with the framing and terminology of non-communicable diseases (NCDs). This paper synthesizes these debates, analysing themes that emerge. The implications are that health professionals may need to be more reflexive about how we classify and discuss diseases and may benefit from other disciplines in addressing the NCD burden. Concepts such as multimorbidity, syndemics, ecosocial theory and health promotion may be particularly useful in this endeavor.
